# Metal ion removal using a low-cost coconut shell activated carbon bioadsorbent in the recovery of lactic acid from the fermentation broth

**DOI:** 10.1186/s40643-023-00672-1

**Published:** 2023-09-01

**Authors:** Wasupon Wongvitvichot, Sitanan Thitiprasert, Nuttha Thongchul, Thanyalak Chaisuwan

**Affiliations:** 1 The Petroleum and Petrochemical College, Chulalongkorn University, Phayathai Road, Wangmai, Pathumwan, Bangkok, 10330 Thailand; 2 Institute of Biotechnology and Genetic Engineering, Chulalongkorn University, Phayathai Road, Wangmai, Pathumwan, Bangkok, 10330 Thailand; 3 Center of Excellence in Bioconversion and Bioseparation for Platform Chemical Production, Chulalongkorn University, Phayathai Road, Wangmai, Pathumwan, Bangkok, 10330 Thailand; 4 The Center of Excellence on Petrochemical and Materials Technology, Chulalongkorn University, Phayathai Road, Wangmai, Bangkok, 10330 Thailand

**Keywords:** Lactic acid recovery, Adsorption, Coconut shell activated carbon, Carboxymethyl cellulose, Thermodynamic and kinetics

## Abstract

Downstream recovery and purification of lactic acid from the fermentation broth using locally available, low-cost materials derived from agricultural residues was demonstrated herein. Surface modification of coconut shell activated carbon (CSAC) was performed by grafting with carboxymethyl cellulose (CMC) using citric acid (CA) as the crosslinking agent. A proper ratio of CMC and CA to CSAC and grafting time improved the surface functionalization of grafted nanostructured CMC-CSAC while the specific surface area and porosity remained unchanged. Lactic acid was partially purified (78%) with the recovery percentage of lactic acid at 96% in single-stage adsorption at room temperature and pH 6 with a 10:1 ratio of cell-free broth to CMC-CSAC bioadsorbent. A thermodynamic study revealed that the adsorption was exothermic and non-spontaneous while the Langmuir isotherm model explained the adsorption phenomena. The results in this study represented the potential of waste utilization as solid adsorbents in green and low-cost adsorption technology.

**Graphical Abstract:**

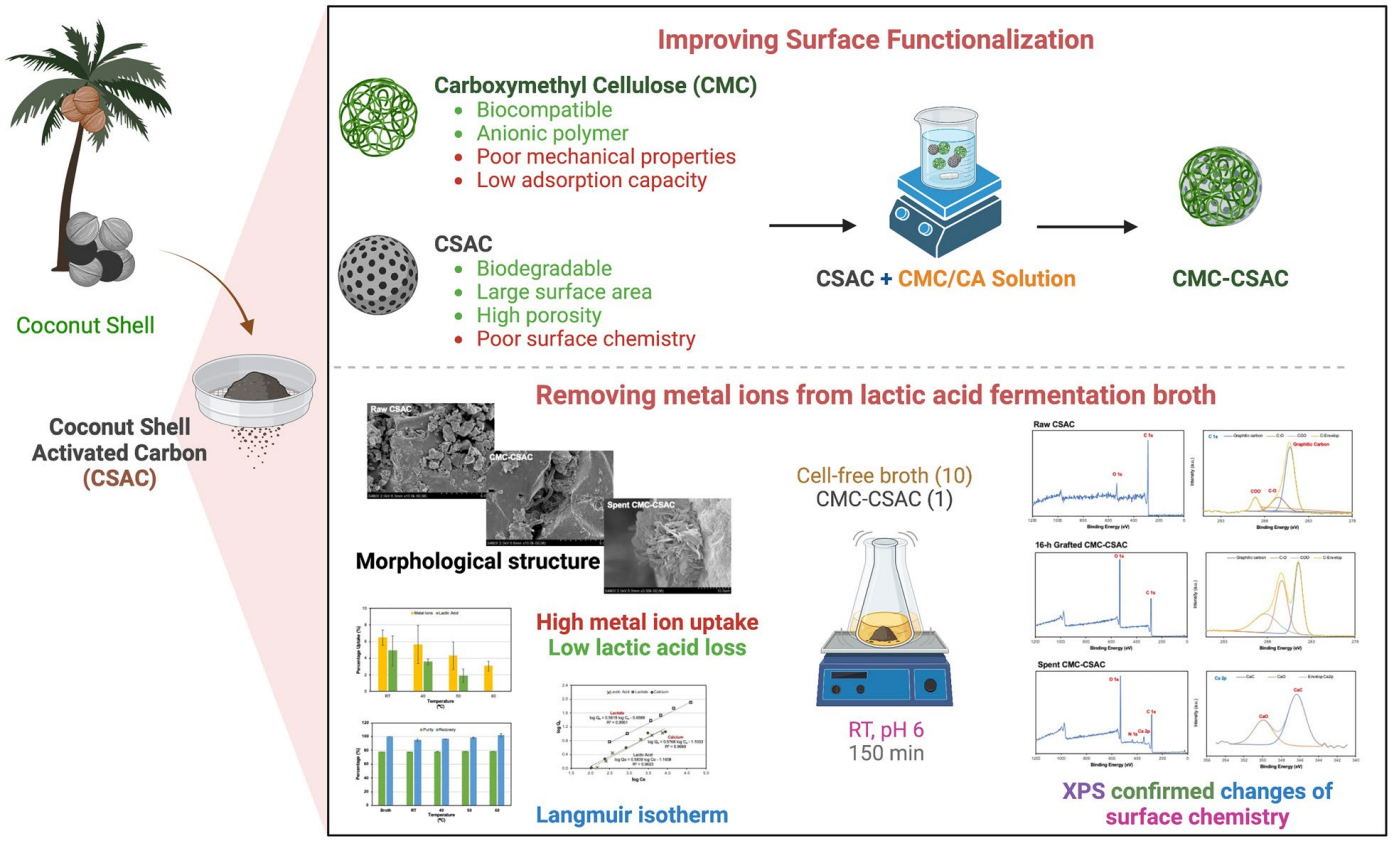

**Supplementary Information:**

The online version contains supplementary material available at 10.1186/s40643-023-00672-1.

## Introduction

The accumulation of petroleum-plastic wastes becomes a major issue of the environmental crisis (Payne et al. 2019). Biomass feedstock has been claimed to be an alternative to petroleum resources for the production of various chemicals via the biorefinery platform because it contains similar C-rich molecules (Thongchul et al. 2022). Lactic acid, 2-hydroxypropanoic acid, is one of the top platform chemicals that has a variety of applications from food and feed to household products, pharmaceuticals, and cosmetics (Ngammuangtueng et al. 2020). In addition, lactic acid can serve as the building block of bio-based plastics including acrylic acid, the precursor of commodity polyvinyl acrylate and biodegradable polylactic acid (Boudrant et al. 2005). Lactic acid is commercially produced by fermentation of carbohydrate-rich substrates under mild operating conditions (Abdel-Rahman et al. 2011). Agricultural residues that contain sugars and starches were reported as a good source of feedstocks in lactic acid fermentation (Pleissner et al. 2017).

The production cost is considered a major factor that drives the market demand for lactic acid for the production of biobased plastics to replace the currently available commodity plastics (Li et al. 2021). The downstream recovery and purification of lactic acid after fermentation contributes to about 40–50% of the total operating cost (Kumar et al. 2020). Several processes have been developed for recovering lactic acid from fermentation broth which requires the key unit operations including precipitation, solvent extraction, adsorption, molecular distillation, esterification, and membrane separation (Li et al. 2021). In the conventional lactic acid production, calcium base was used for pH control during fermentation which resulted in calcium lactate as the final product. Free lactic acid was recovered from the fermentation broth by acidification using H_2_SO_4_. CaSO_4_ together with the cell biomass, also known as the biosludge, was the major byproduct obtained during the primary recovery step that required further waste treatment (Min et al. 2011). Solvent extraction is typically used in the recovery of lactic acid from the cell-free fermentation broth. This process is not environmentally safe due to the consumption of toxic chemicals. Membrane separation, on the other hand, does not require toxic chemicals, and it is considered a green and low-energy demand technology and is easy to scale up. The major drawback of membrane technology is the short lifetime of the membrane due to fouling. This results in high operating cost. Electrodialysis uses the electric field instead of the pressure as the driving force which enhances membrane separation efficiency, but it is an energy intensive process (Boonkong et al. 2009; Kumar et al. 2020). Reactive distillation together with the novel catalysts yields highly pure lactic acid but it is also an energy intensive process which requires high investment cost (Xu et al. 2018). Esterification is claimed to be the technique that yields more than 95% pure lactic acid. However, this technique cannot be used alone to recover and purify lactic acid. Typically, purification of lactic acid by esterification requires other unit operations including fractionation and hydrolysis. These process steps also require high energy consumption and generate a large amount of wastewater (Li et al. 2021).

Compared with the key techniques mentioned earlier, adsorption provides the advantages as it is a low energy, chemical process that provides sufficiently high purity and recovery percentages. The operation is simple and easy to scale up. Previous studies reported that cationic and anionic ion exchange resins such as Amberlite IRA-96, Amberlite IRA-67, and Amberlite IRA-120 were used to recover organic acid (Bayazit et al. 2011; Bishai et al. 2015; Zaini et al. 2019). Adsorption using polymeric ion exchange resins to recover the desirable product consists of 5 major steps including pretreatment of the resins, adsorption, washing, desorption, and resin regeneration. Each step requires a large amount of acid, base, enzyme, and water. This eventually increases the wastewater treatment load (Bishai et al. 2015; Gonzalez et al. 2006).

Besides the polymeric ion exchange resins, other materials such as activated carbon can be used as the adsorbent. Activated carbon is a low-cost material derived from plant biomass and agricultural residues. The major characteristics of activated carbon include its biodegradability, large surface area, and high porosity but poor surface chemistry (Thubsuang et al. 2023). This material is frequently used for removal of organic pollutants, dyes, and heavy metals in waste treatment (Du et al. 2014). Carboxymethyl cellulose (CMC), on the other hand, is a biocompatible anionic polymer that has a good surface chemistry, but it has poor mechanical properties and low adsorption capacity (He et al. 2019). In this study, we aimed to develop a simple adsorption process using low-cost materials as the adsorbents to remove the metal impurities from the cell-free fermentation broth and therefore recover lactic acid. To achieve this goal, the surface modification of coconut shell activated carbon (CSAC) was conducted by grafting with CMC with citric acid (CA) as the crosslinking agent. The kinetic and thermodynamic processes were investigated under different operating conditions to explain the adsorption of metal ions and lactic acid on the CSAC adsorbents. The results in this study provided the insights on adsorption technology using the locally available, low-cost materials as adsorbents.

## Materials and methods

### Chemicals and materials

CMC and CA were purchased from Siam Bakery Land Company Limited (Bangkok, Thailand). Lactic acid solution (LA), 88% *v*/*v*, was purchased from Chemipan Corporation Company Limited (Bangkok, Thailand). Hydrochloric acid (HCl) 37% *v*/*v*, sulfuric acid (H_2_SO_4_) 98% *v*/*v*, and sodium hydroxide (NaOH) were purchased from RCI Labscan Company Limited (Bangkok, Thailand). Calcium lactate pentahydrate (CaLA) was purchased from Merck Company Limited (Bangkok, Thailand). All chemicals were used without further purification. CSAC with a mesh size of 12 × 40 and 3.0% ash was obtained from Carbokarn Company Limited (Thailand). CSAC was purified with deionized water (DI) until the conductivity was lower than 50 µS/cm.

### Feed preparation

The fermentation broth was obtained from the cultivation of *Terrilactibacillus laevilacticus* SK5-6 in a 5 L stirred fermenter following the protocol reported in Prasirtsak et al. (2019) and Thitiprasert et al. (2017). An active 24 h glucose yeast extract peptone slant was used to prepare the bacterial suspension. The bacterial suspension (1% inoculum size) was inoculated in a preculture vessel containing the preculture medium. The preculture medium contained (per liter) 10 g glucose, 15 g yeast extract, 4 g NH_4_Cl, 0.5 g KH_2_PO_4_, 0.5 g K_2_HPO_4_, 5 g CaCO_3_, and 20 mL salt solution. The compositions of the salt solution consisted of (per 10 mL) 400 mg MgSO_4_·7H_2_O, 20 mg MnSO_4_·5H_2_O, 20 mg FeSO_4_·7H_2_O, and 20 mg NaCl. The preculture flask was incubated at 37 °C, 200 rpm for 5 h. The preculture broth was then transferred into the 5 L stirred fermenter containing 2.25 L preculture medium (10% inoculum). The fermenter was controlled at 37 °C, 300 rpm, 1.0 vvm air for 3 h. After that, 1 L of concentrated glucose solution (350 g/L) was added into the fermenter to obtain 100 g/L initial glucose concentration. Sterile CaCO_3_ powder (280 g) was added into the fermenter as the buffering power during lactic acid fermentation. The aeration was stopped. The fermentation was continued at the same temperature and agitation rate until glucose depletion. The fermentation broth was harvested and subsequently centrifuged and filtered to remove the cell biomass. The cell-free fermentation broth compositions were determined. It mainly contained ~ 80–100 g/L lactate, ~ 0–26 g/L glucose, 16.25 g/L Ca^2+^, 0.17 g/L K^+^, 0.19 g/L Mg^2+^, 0.07 g/L Na^+^, 0.02 g/L Fe^2+^, 1.05 g/L Cl^−^, and 0.20 g/L SO_4_^2−^ (Additional file 1: Table S1).

### Preparation and characterization of coconut shell activated carbon adsorbent

The surface of CSAC adsorbent was modified before use. CMC was prepared by dissolving in 10 mL DI water at various concentrations (1–2% *w*/*v*). Citric acid was added into CMC solution at various concentrations (5–20% *w*/*v*). The solution was stirred at room temperature until it became homogeneous. Afterwards, 5 g CSAC was added into the solution and the mixture was stirred for another 30 min. The mixture was incubated at 120 °C to allow complete esterification. The effect of incubation time (8–48 h) on CSAC surface grafting was also investigated. Later, the mixture was washed thoroughly with DI water until the conductivity was lower than 50 µS/cm and dried at 120 °C overnight to obtain the grafted adsorbent (CMC-CSAC).

The morphology and surface chemistry of the CMC-CSAC adsorbent were observed by field-emission scanning electron microscopy (FE-SEM) and x-ray photoelectron spectrophotometry (XPS). The Brumaruer-Emmett-Teller (BET) nitrogen adsorption/desorption method was used to determine the specific surface area and pore size distribution. The total pore volume and micropore at a relative pressure of 0.995 were analyzed using the t-plot method.

### Ion adsorption on CSAC adsorbent

A variety of CMC-CSAC adsorbents (5 g) previously prepared as previously stated was mixed with 50 mL cell-free lactic acid fermentation broth (pH 6). The mixture was stirred at room temperature for 150 min before filtering through Whatman filter paper No.42. The liquid portion was kept for analyses of metal ions and lactic acid concentration using atomic adsorption spectrophotometry (AAS), high performance liquid chromatography (HPLC), and ion chromatography (IC).

The optimal contact time was determined. Five g of CMC-CSAC adsorbent grafted with 2% CMC and 20% CA was mixed with 50 mL cell-free lactic acid fermentation broth at room temperature. The contact time was varied (0–24 h). Later, the mixture was filtered through Whatman filter paper No.42. The liquid portion was kept for analyses of metal ions and lactic acid. The effect of the initial pH of the fermentation broth and operating temperature on ion adsorption onto CMC-CSAC was studied. After harvesting, lactic acid fermentation broth had an initial pH of 6. The pH was adjusted to 2 by adding 10 M H_2_SO_4_. The adsorption capacity was also investigated by varying the ratio of lactic acid fermentation broth (L) to solid CMC-CSAC adsorbent (S) from 2.5 to 20.

### Determination of adsorption mechanism and capacity on CMC-CSAC adsorbent

One g of CMC-CSAC grafted with 2% CMC and 20% CA for 16 h was mixed with 10 mL lactic acid and calcium lactate (CaLA) solution (pH 6) at various concentrations (${C}_{0}$) (0.25–10 g/L lactic acid and 0.5–25 g CaLA) at room temperature for 24 h until equilibrium. The liquid sample from the mixture was filtered and collected for analyses of free lactic acid, Ca^2+^ and lactate ions at equilibrium (${C}_{\mathrm{e}}$). The equilibrium ion adsorption capacity (${Q}_{\mathrm{e}}$) was determined by Eq. (1).

1 $${Q}_{\mathrm{e}}=\frac{\left({C}_{o}-{C}_{\mathrm{e}}\right)V}{m}$$

where *m* was the amount of CMC-CSAC in g used and *V* was the volume of lactic acid and CaLA solution in mL.

Three different isotherm models including Freundlich, Langmuir, and Temkin were fitted to the experimental data to determine the adsorption mechanism and capacity. These models were described in Eqs. (2, 3, 4).

Freundlich:

2 $${Q}_{\mathrm{e}}={\mathrm{K}}_{\mathrm{F}}{C}_{e}^{1/\mathrm{n}}$$

Where K_F_ (L^1/n^mg^1−1/n^/g) and n were constants.

Langmuir:

3 $${Q}_{\mathrm{e}}=\frac{{Q}_{\mathrm{m}}{K}_{L}{C}_{e}}{1+{K}_{L}{C}_{e}}$$

Where *K*_*L*_ (L/mg) was the ratio of the adsorption rate to the desorption rate and *Q*_m_ (mg/g) was the maximum adsorption capacity estimated by the Langmuir model.

Temkin:

4 $${Q}_{\mathrm{e}}=\frac{\mathrm{R}T}{\mathrm{b}}\mathrm{ln}(\mathrm{A}{C}_{e})$$

Where A (L/mg) and b (J/mol) were constant, R was the universal gas constant (8.314 J/mol K) and *T* was the absolute temperature (K).

### Analytical methods

High performance liquid chromatography (HPLC) was used to analyze the concentration of lactic acid and glucose in the solution. Initially, the solution was filtered through a hydrophilic polytetrafluoroethylene membrane and diluted with double deionized water. Then, 15 μL of diluted particle-free sample was automatically injected (Shimadzu-SIL-20A HT, Japan) into an organic acid column (Bio-Rad, Aminex HPX-87H ion exclusion organic acid column; 300 mm × 7.8 mm, Bio-Rad, USA). The sample was maintained at 45 °C in the column oven (Shimadzu-CTO-20A, Japan) and eluted with 5 mM H_2_SO_4_ at a flow rate of 0.6 mL/min (Shimadzu-LC-20A, Japan). A refractive index detector (Shimadzu-RID-20A, Japan) was used to detect lactic acid and glucose. To determine the concentration from the peak areas, standards containing lactic acid and glucose at the concentration of 0–2 g/L were injected as references.

An atomic adsorption spectrophotometer (GBC Scientific Equipment Pty Ltd) was used to determine the concentration of metal ions including Ca^2+^, K^+^, Mg^2+^, Na^+^, Fe^2+^, and Mn^2+^ using the GBC Avant aver. 2.02 software.

An x-ray photoelectron spectrophotometer (XPS, Kranos Axis Ultra DLD) was used to measure the surface potential of CSAC adsorbents. It was equipped with the monochromatic A1 Kα X-ray source (anode HT, 15 kV) in the pressure chamber (lower than 5 × 10^–7^ torr). Forty eV was used for the narrow scan and the binding energy was calibrated at the C–C peak of C 1s at 284.6 eV.

A field-emission scanning electron microscope (FE-SEM, Hitachi S-4800) was used to observe the morphology of CSAC adsorbents. The specimens were prepared by affixing the samples onto a metal stub-supported carbon tape. The specimens were coated with platinum under a current of 20 mA using an ion-sputter (Hitachi E-1010).

The Brumaruer-Emmett-Teller (BET) nitrogen adsorption/desorption method was used to determine the specific surface area and the pore size distribution (Quantachrome Autosorb 3 XR). The quenched solid density functional theory (QSDFT) equilibrium model was applied with a relative pressure (P/P_O_) from 1 × 10^–5^ to 0.995. The sample with the approximate weight of 0.05 g was degassed at 150 °C overnight. The total pore volume and micropores were analyzed using a relative pressure of 0.995 and the t-plot method.

### Calculation

Recovery and purity percentages of lactic acid were determined using the following equations.

5 $$\% \mathrm{Recovery}=\frac{\mathrm{Amount\,of\,lactic\,acid\,in\,the\,solution\,after\,adsorption}\,(g)}{\mathrm{Amount\,of\,lactic\,acid\,in\,the\,solution\,before\,adsorption} (g)}\times 100$$

6 $$\% \mathrm{Purity}=\frac{\mathrm{Amount\,of\,lactic\,acid\,in\,the\,solution}\,(g)}{\mathrm{Amount\,of\,lactic\,acid\,in\,the\,solution} \left(g\right)+ \mathrm{Amount\,of\,impurities\,in\,the\,solution}\,(g)}\times 100$$

The percentage of ion uptake onto the surface of CMC-CSAC adsorbent was calculated by Eq. (7).

7 $$\% \mathrm{Uptake}=\frac{\mathrm{Amount\,of\,ion\,before\,adsorption}\,\left(g\right)-\mathrm{Amount\,of\,ion\,after\,adsorption}\,(g)}{\mathrm{Amount\,of\,ion\,before\,adsorption}}\times 100$$

## Results and discussion

The cell biomass and large particles including proteins that remained in the harvested fermentation broth were removed by centrifugation, microfiltration, and ultrafiltration. The cell-free fermentation broth was later used in lactic acid recovery by eliminating the remaining metal ions, anions, and glucose. In this study, CMC-CSAC adsorbent composing of 3 components including CSAC, CMC, and CA was mainly used for removing metal ions from the cell-free fermentation broth. CSAC was used as the solid support that allowed the adsorption process to occur. Besides, CSAC adsorbed both polar and non-polar molecules (Anirudham et al. 2011; Rivera-Utrilla et al. 2009). CMC provided the active sites to adsorb polar molecules and CA as a grafting agent allowed crosslinking of CMC and CSAC (Yang et al. 2011; Zhou et al. 1995).

### Surface modification of CSAC to improve metal ion adsorption

**Fig. 1:**
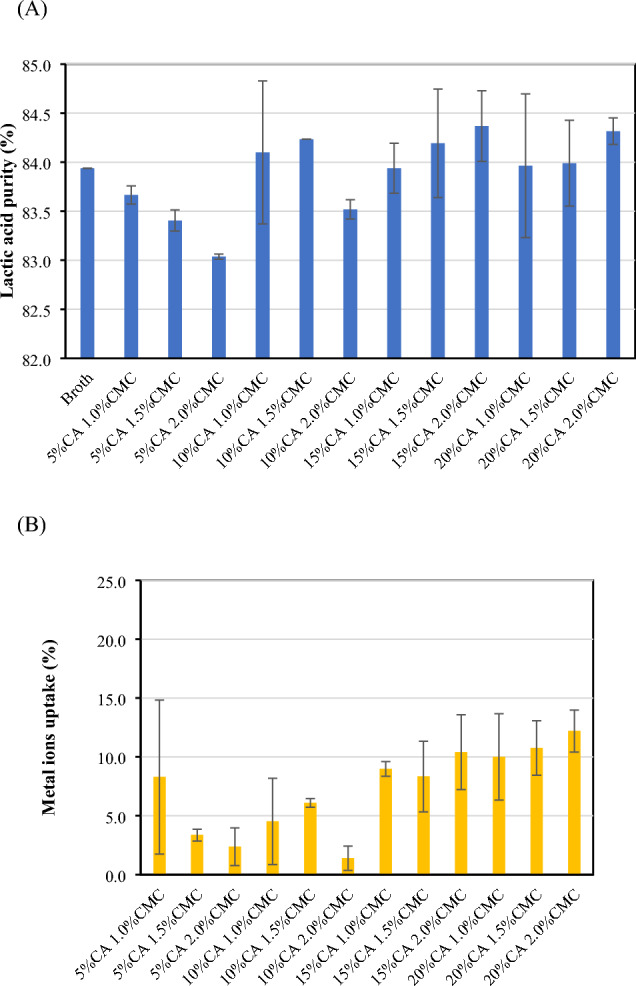
Percentages of lactic acid purity (**A**) and metal ion uptake (**B**) of lactic acid fermentation broth (50 mL) after soaking in CMC-CSAC adsorbents (5 g) with varied concentrations of CMC and CA at room temperature for 150 min

Figure 1 shows the percentages of lactic acid purity and the metal ion uptake after adsorption in different CMC-CSAC adsorbents. CA concentration of 5% *w*/*v* caused the decrease in lactic acid purity (Fig. 1A) after adsorption due to poor metal ion uptake (Fig. 1B). Increasing CA concentration enhanced lactic acid recovery as observed from the increasing metal ion uptake and purity percentage of lactic acid compared with that in the cell-free fermentation broth (83.94%). The maximum CMC concentration was 2% *w*/*v* due to its solubility in water. The purity percentage of lactic acid after adsorption onto grafted CMC-CSAC (20% CA and 2.0% CMC) was the highest with the corresponding recovery percentage of 90.03 and 12.19% metal ion uptake.

The carboxylic functional group in CMC and CA was responsible for the differences in adsorption performance of grafted CMC-CSAC at various concentrations of CA and CMC. The carboxylic functional group typically found in weak anion exchange resins played a role in metal ion removal as it bound with the positively charged ions. Due to the 3 carboxylic groups in CA structure, it also acted as the crosslinker at the optimized concentration of both CA and CMC resulting in the grafted CMC-CSAC adsorbent with more active sites that promoted metal ion adsorption (Fig. 1B) (Eltaweil et al. 2020). The decrease in purity percentage of lactic acid in the solution after adsorption was explained by the electrostatic interaction between the carboxylic group in lactic acid and the hydroxyl group in CMC and CSAC. This resulted in the loss of lactic acid from the fermentation broth and eventually lowered lactic acid purity. The hydroxyl group in the main chain of CMC and the surface of CSAC had a positive charge whereas the carboxylic group in lactic acid had a negative charge with the respective pKa values of 12 and 3.8 (Bialik et al. 2016). This caused lactic acid adsorption onto the grafted CMC-CSAC at a low CA concentration of 5% *w*/*v*. The number of hydroxyl groups of the pyranose ring at the main chain of CMC and the surface of CSAC was decreased with the increasing concentration of CA and CMC during grafting. The surface charge of CMC-CSAC adsorbent became more negative from the increasing number of carboxylic groups when compared with that of the raw CSAC, resulting in increasing metal ion uptake (Fig. 1B) (Yang et al. 2011).

**Fig. 2:**
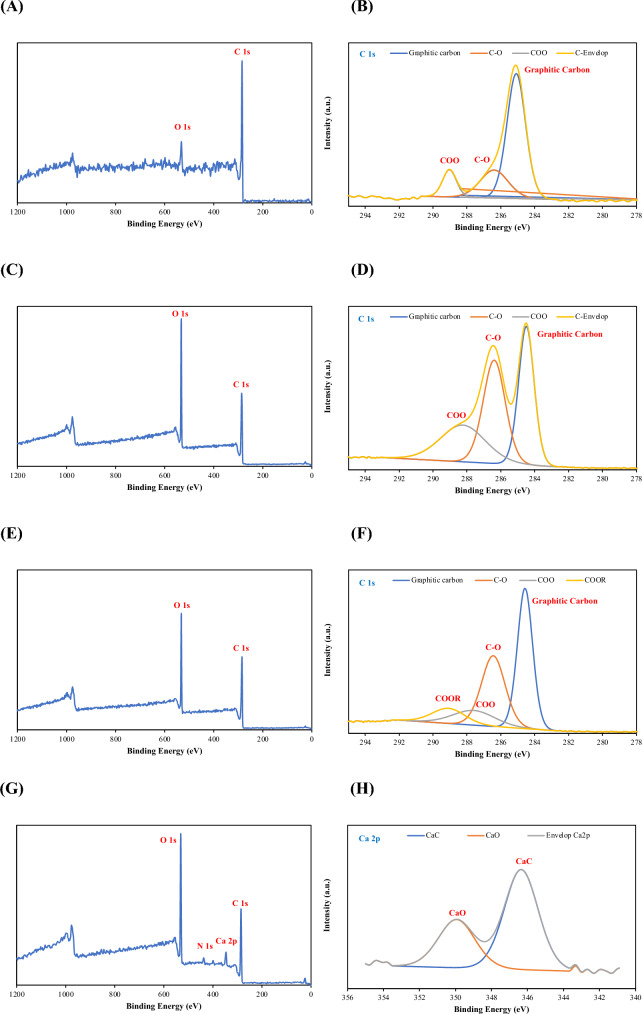
XPS analysis results showing 1. The wide-scan spectra of raw CSAC (**A**), 16-h grafted CMC-CSAC (**C**), 48-h grafted CMC-CSAC (**E**) and spent 16-h grafted CMC-CSAC (**G**); and 2. The narrow-scan spectra of raw CSAC (**B**), 16-h grafted CMC-CSAC (**D**), 48-h grafted CMC-CSAC (**F**), and spent 16-h grafted CMC-CSAC (**H**)

This evidence was confirmed by XPS results (Fig. 2). The XPS wide-scan spectra of the raw CSAC, 16-h grafted CMC-CSAC, and 48-h grafted CMC-CSAC exhibited C 1s at 284.6 eV and O 1s at 532.5 eV (Fig. 2A, C, E) (Zhou et al. 2007). The 3 functional components, including the graphitic carbon at 285.0 eV, the hydroxyl or ether (CO) at 286.4 eV, and the carboxyl (COO^−^) at 288.5 eV (Fig. 2B, D, F), were assigned by the narrow-scan spectra. When CSAC was grafted with CMC and CA, the number of functional carboxyl and ether groups were increased (Table 1). With more negatively charged functional groups at the surface of the adsorbent, the metal ion adsorption improved (Kadirvelu et al. 2001; Zhang et al. 2014). Besides the C 1s and O 1s peaks, the N 1s at 396.8 eV and the Ca 2p at 345.9 eV and 349.4 eV appeared in the XPS spectra of the spent CMC-CSAC after adsorption (Fig. 2G). The chemical bonds of Ca^2+^ to the CMC-CSAC adsorbent, namely Ca_2_C at 344.5 eV and CaO/CaCO_3_/Ca(OH)_2_ at 347.6 eV (Fig. 2H), were shown in the Ca 2p spectra (Ghods et al. 2011).

**Table 1:** XPS quantitative analysis of the surfaces of CSAC, CMC-CSAC, and spent CMC-CSAC. CMC-CSAC was prepared by grafting CSAC with 2% CMC and 20% CA at different times

Grafting time (h)	Elemental, %	Deconvolution, %					
		C 1s				Ca 2p	
	Graphitic carbon	CO	COO	COOR	Ca_2_C	Ca_2_C	CaO
8	42.77	39.08	18.15	ND	ND	ND	ND
16	35.35	37.38	27.28	ND	ND	ND	ND
24	26.49	58.96	8.62	5.94	ND	ND	ND
32	24.03	55.23	4.05	16.69	ND	ND	ND
48	43.26	33.87	12.68	10.19	ND	ND	ND
Raw CSAC	77.80	14.18	8.20	ND	ND	ND	ND
Spent CMC-CSAC	54.63	16.46	6.11	ND	22.81	27.37	72.63

*ND* not detected

**Fig. 3:**
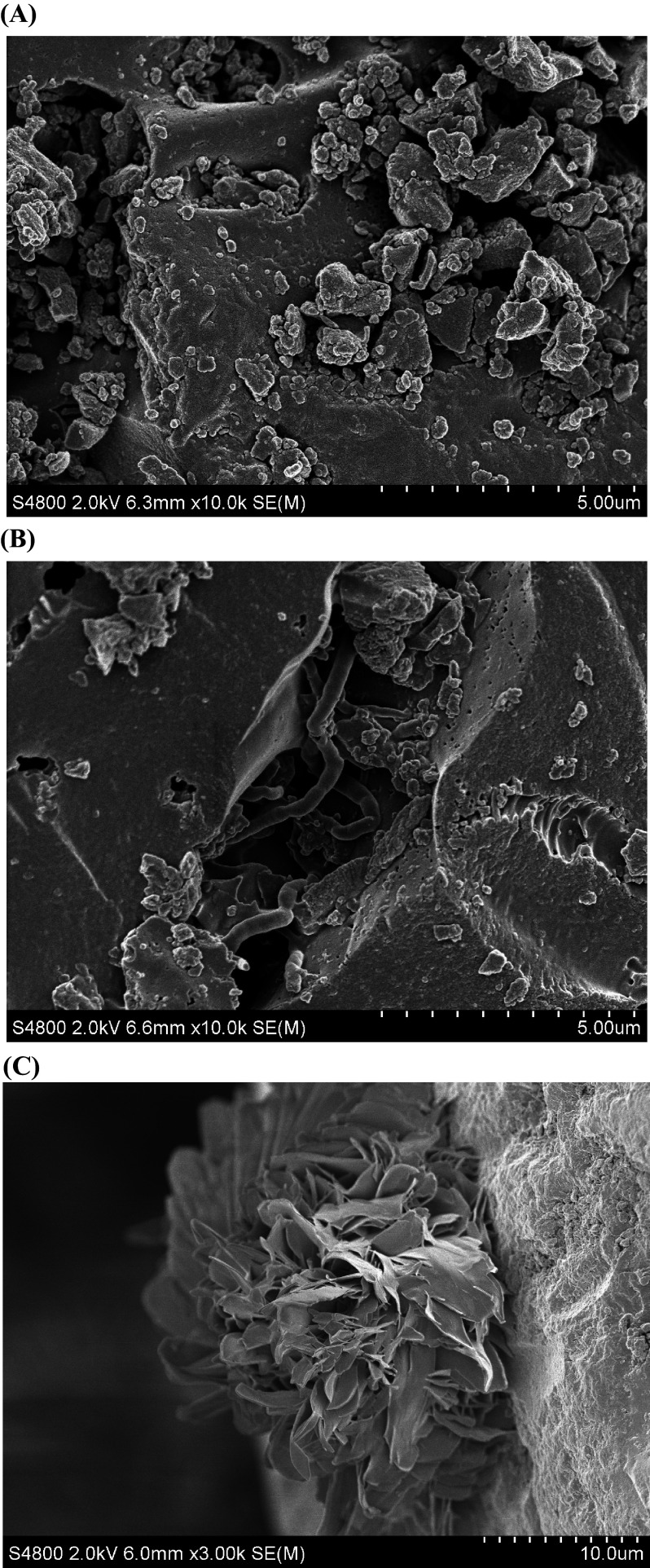
SEM micrographs showing the morphology of **A** CSAC, **B** CMC-CSAC, and **C** spent 16-h grafted CMC-CSAC after adsorption

The SEM micrographs of CSAC adsorbents revealed surface grafting and metal ion adsorption. The raw CSAC was grafted with 2.0% CMC and 20% CA for 16 h. Compared with the rough surface of the raw CSAC shown in Fig. 3A, the rough surface of CSAC with the rod-like shape CMC appeared on the micrograph of the grafted CMC-CSAC (Fig. 3B) (Upadhyaya et al. 2014). A plate-like shape crystal of calcium lactate was observed on the structure of CMC-CSAC indicating the occurrence of metal adsorption (Fig. 3C) (Tesfamariam et al. 2022). The morphology characterization confirmed that surface modification of CSAC using the proper ratio of CA to CMC enhanced the adsorption of metal ions and the recovery of lactic acid.

### Grafting time improved adsorption performance

**Fig. 4:**
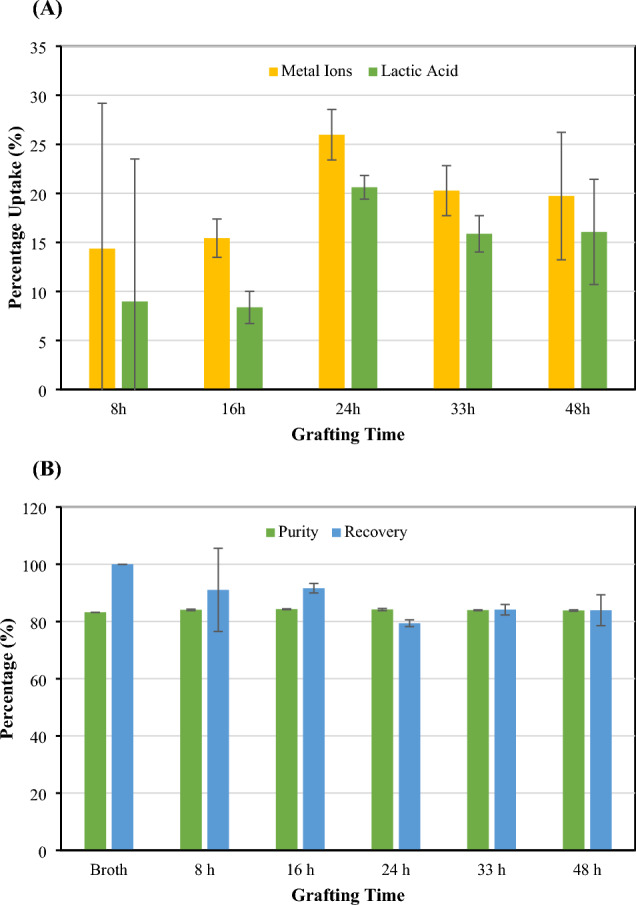
Effect of grafting time during the preparation of CMC-CSAC adsorbent on **A** the uptake of metal ions and lactic acid and **B** lactic acid purity and recovery percentages in the solution after purification. CMC-CSAC was prepared by mixing 10 mL solution containing 2% CMC and 20% CA with 5 g CSAC before grafting for different durations. The adsorption was carried out by soaking 5 g CMC-CSAC in 50 mL lactic acid fermentation broth (pH 6) at room temperature for 150 min

The effect of grafting time during the preparation of the CMC-CSAC adsorbent on metal ion adsorption was investigated. It was observed that both metal ions and lactic acid were adsorbed onto the CMC-CSAC surface. Grafting time affected the percentage uptake of both metal ions and lactic acid (Fig. 4A). Increasing the grafting time from 8–16 to 24–48 h caused more metal ions and lactic acid to be adsorbed onto the CMC-CSAC surface. This eventually led to the reduction of lactic acid recovery in the solution after adsorption while the purity did not change (Fig. 4B). The difference in ion adsorption capacity of CMC-CSAC prepared at different grafting times can be explained by the grafting mechanism. The mechanism began with the formation of CA cyclic anhydride due to heating during the condensation reaction of the carboxylic group. The cyclic anhydride reacted with the hydroxyl group of CMC and CSAC in the esterification reaction, which resulted in an ester bond between CMC and the CSAC surface (Mali et al. 2017). The proper surface modification of CSAC was obtained with the proper reaction time (grafting time). A longer grafting time promoted the crosslinking between CMC chains as observed from the XPS results (Table 1) (Zheng et al. 2015). It was observed that the number of functional carboxylic (COO^−^) groups was increased with the increasing grafting time from 8 to 16 h. A grafting time more than 16 h led to a decrease in the amount of COO^−^ and an increase in the amount of carboxylate ester (COOR) (Liu et al. 2010; Mazouzi et al. 2019; Swiatkowski et al. 2004). COOR was claimed to be responsible for lactic acid adsorption. This coincided with the percentage loss of lactic acid shown in Fig. 4A. Therefore, from the results shown in Fig. 4, 16 h was sufficient for grafting of CMC and CSAC by CA. This resulted in sufficiently high metal ion uptake (15.43% uptake) with a lower loss of lactic acid (8.97% loss) and 91.03% recovery of the solution.

### The effect of pH, contact time, and adsorbent loading on adsorption performance

**Fig. 5:**
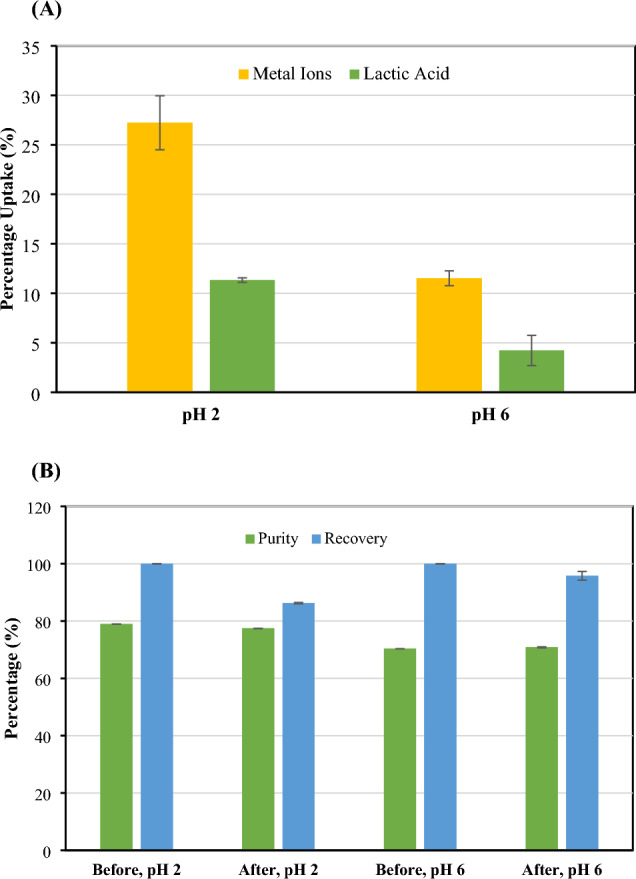
Effect of the initial pH of cell-free lactic acid fermentation broth (50 mL) on **A** the uptake of metal ions and lactic acid on 16-h grafted CMC-CSAC (5 g) and **B** lactic acid purity and recovery percentages in the solution after adsorption. The operation condition was at room temperature for 150 min. CMC-CSAC was prepared by mixing 10 mL solution containing 2% CMC and 20% CA with 5 g CSAC before grafting for 16 h

The pKa of lactic acid is 3.8 (Pradhan et al. 2017). Lactic acid becomes more negatively charged when the pH is greater than its pKa, whereas it is more acidic (higher H^+^ concentration) at a pH lower than its pKa. During lactic acid fermentation, CaCO_3_ was sufficiently added into the fermentation broth to maintain an optimal pH. At the end of the fermentation, the pH of the fermentation broth was 6.12. The fermentation broth therefore mainly contained 2 lactate species including calcium lactate and free lactic acid. The concentration of lactate species present in the fermentation broth was 100.54 g/L, the concentration of metal ions was 16.70 g/L and that of the remaining glucose was 25.82 g/L, with a corresponding initial lactic acid purity of 70.28%. To adjust the pH of the fermentation broth to 2 before adsorption by CMC-CSAC, the fermentation broth was acidified using 10 M H_2_SO_4_. The acidification allowed gypsum (CaSO_4_) precipitation which resulted in an increase in the purity of the clarified broth from 70.28 to 78.92% (Min et al. 2011). Figure 5 depicted the effect of the initial pH of the cell-free fermentation broth on the adsorption process. At the acidic pH (pH 2), more metal ions and lactic acid were adsorbed onto the negatively charged surface of CMC-CSAC due to the stronger electrostatic interaction. Whereas at pH 6, the fermentation broth was more negatively charged. This resulted in electrostatic repulsion (Fig. 5A) (Dai et al. 2018). On the other hand, the hydrogen bonding and the acid–base interaction were claimed to be responsible for the percentage loss of lactic acid when the pH was lower than the pKa (Cao et al. 2002). This resulted in the lower recovery percentage of lactic acid at pH 2 compared with that at pH 6 while the purity percentage did not change (Fig. 5B). Therefore, the optimal cell-free fermentation broth pH that favored metal ion adsorption and lactic acid recovery was 6.

**Fig. 6:**
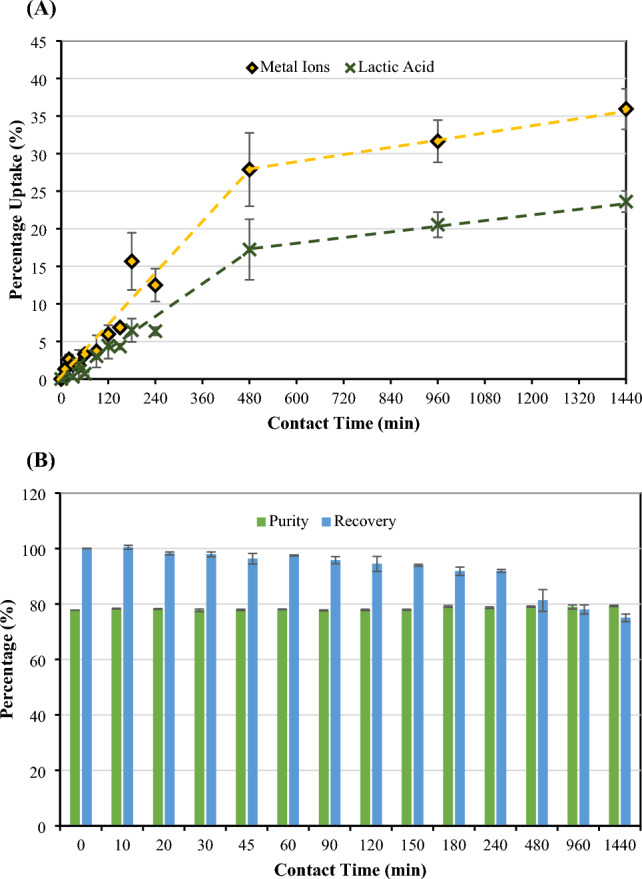
Effect of contact time on **A** adsorption capacity of metal ions and lactic acid and **B** lactic acid purity and recovery in the solution after adsorption. The 16-h grafted CMC-CSAC (5 g) was soaked in 50 mL lactic acid fermentation broth (pH 6) at room temperature with varied contact time

The effect of contact time on the separation performance of CMC-CSAC was observed. Figure 6A displayed the adsorption of metal ions and lactic acid from the cell-free fermentation broth (50 mL) onto 16-h grafted CMC-CSAC (5 g). The adsorption was carried out at room temperature and pH 6. From the percentage uptake, it was clear that metal ions were strongly bound to the surface of CMC-CSAC compared with lactic acid. The longer the contact time was, the more metal ions and lactic acid CMC-CSAC adsorbed. During the first 480 min, both metal ions and lactic acid were rapidly bound onto the adsorbent surface as seen from the increase in percentage uptake to 27.87% and 17.24%, respectively. Later, the adsorption capacity decreased. It was observed that the purity percentage of lactic acid did not significantly change (approximately 77–79%) with the increasing contact time, whereas the recovery percentage continuously dropped (from 100% at 0 min to 93.96% at 150 min and 81.27% at 480 min when approaching the adsorption equilibrium) (Fig. 6B). Based on the recovery performance and economical operational time, 150 min adsorption gave a good metal ion uptake (6.86%) with a low percentage of lactic acid loss (4.32%) (Anirudhan and Sreekumari 2011; Tao et al. 2015). Therefore, the subsequent experimental runs were carried out at this condition.

**Fig. 7:**
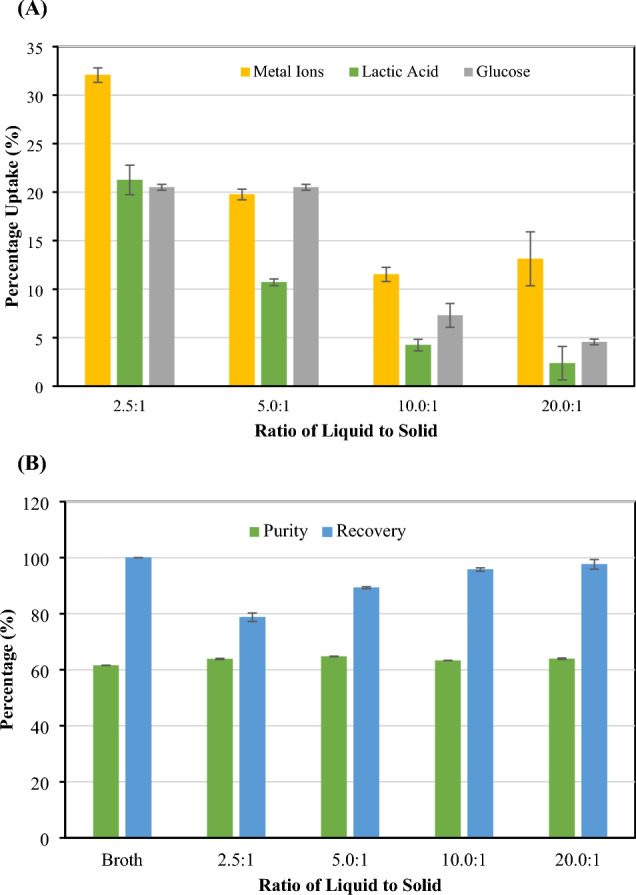
Effect of CMC-CSAC loading on **A** adsorption capacity of metal ions, glucose, and loss of lactic acid and **B** lactic acid purity and recovery in the solution after adsorption. The 16-h grafted CMC-CSAC (varied amount in g) was soaked in 50 mL lactic acid fermentation broth (pH 6) at room temperature for 150 min

The effect of CMC-CSAC loading on adsorption of metal ions and glucose as well as lactic acid recovery was observed in the batch experiment with varied ratios of liquid fermentation broth to solid CMC-CSAC adsorbent (Fig. 7). A larger surface area was acquired when more adsorbent was used to adsorb ions in a certain broth volume (Pellera et al. 2012; Ren et al. 2016). In this study, the percentage uptake of both metal ions, glucose, and lactic acid was increased when the ratio of liquid fermentation broth to solid CMC-CSAC was decreased from 10.0:1 to 2.5:1 while the percentage uptake of metal ions, glucose, and lactic acid was not much different between the ratios of 10.0:1 and 20.0:1 (Fig. 7A). Besides metal ions, CMC-CSAC adsorbent could also adsorb glucose. At pH 6 (the pH of the fermentation broth, glucose became positively charged. This supported the electrostatic attraction between glucose and CMC-CSAC (Feng et al. 2013). It was found that at the ratios of 2.5:1 and 5.0:1, more glucose was adsorbed (~ 20% uptake) when soaking CMC-CSAC into the fermentation broth (Fig. 7A). Glucose adsorption drastically dropped when increasing the ratio to 10:1 (7.30% uptake). At the ratio of 20.0:1, the lowest glucose adsorption was observed. Lactic acid purity (~ 64%) in the cell-free fermentation broth after adsorption seemed to not change while the recovery percentage dropped when the ratio was decreased from 10.0:1 (95.77%) to 5.0:1 (89.30%) (Fig. 7B). It was clear that using excess adsorbent resulted in lactic acid loss and cost contribution. Therefore, in the subsequent runs, the adsorption was carried out using a 10.0:1 ratio of cell-free fermentation broth to CMC-CSAC.

### Thermodynamic study of ion adsorption onto the CMC-CSAC surface

**Fig. 8:**
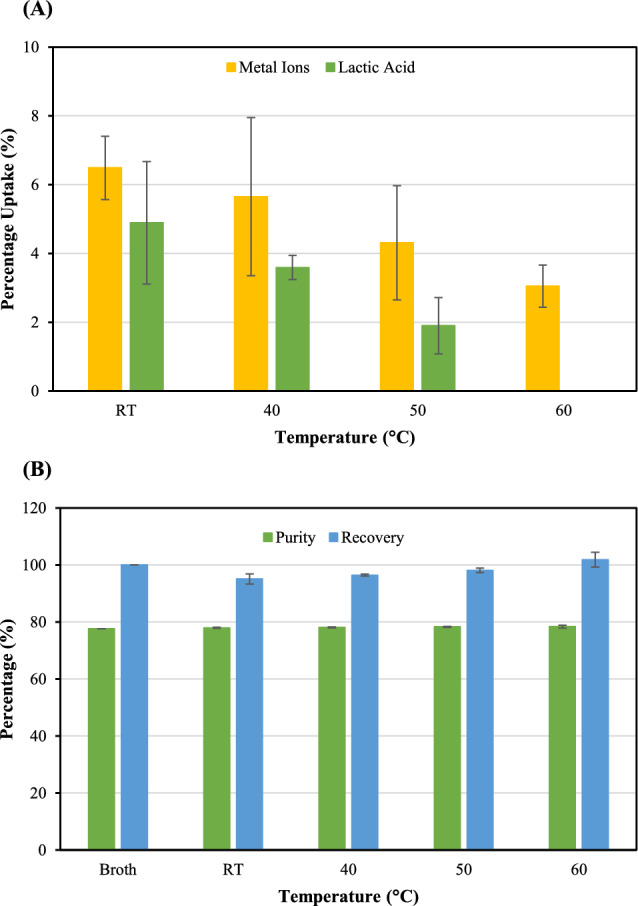
Temperature effect on **A** adsorption of metal ions and lactic acid and **B** the purity and recovery percentage of lactic acid in the solution after adsorption. The 16-h grafted CMC-CSAC (5 g) was soaked in 50 mL lactic acid fermentation broth (pH 6) at varied temperature for 150 min

It was reported that the effect of temperature on the adsorption process as the temperature changed typically led to changes in the structure of the binding site and the interaction between the adsorbent and the adsorbate (Ren et al. 2016). In this study, the adsorption was carried under different temperatures from room temperature (~ 25 °C) up to 60 °C. When the temperature increased, the percentage uptake of both metal ions and lactic acid decreased (Fig. 8A). Lactic acid did not adsorb onto the surface of CMC-CSAC at 60 °C. Increasing the operating temperature did not affect the purity and recovery of lactic acid after adsorption (Fig. 8B). From the results shown in Fig. 8, the removal of metal ions and the recovery of lactic acid could be achieved at room temperature to maintain low energy input.

The thermodynamic parameters of the adsorption of metal ions and lactic acid onto the surface of CMC-CSAC were determined to characterize the adsorption mechanism using the van’t Hoff plot and the Gibbs Free Energy equation.

van’t Hoff Equation

8 $$\mathrm{ln}\frac{{Q}_{\mathrm{e}}}{{C}_{\mathrm{e}}}=-\frac{\Delta H}{\mathrm{R}T}+\frac{\Delta S}{\mathrm{R}}$$

Gibbs Free Energy

9 $$\Delta G=\Delta H-T\Delta S$$

**Fig. 9:**
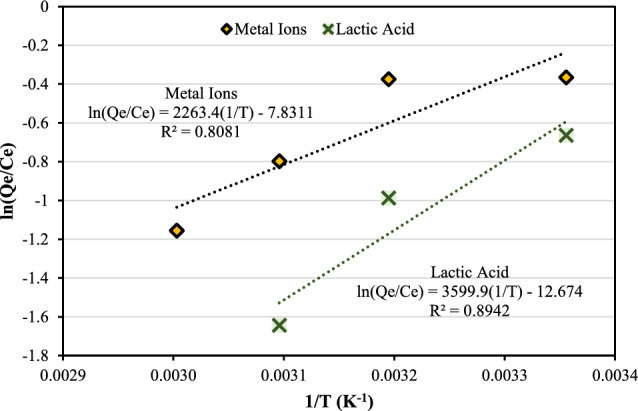
The van’t Hoff plot for determination of the changes in enthalpy and entropy in the adsorption of metal ions and lactic acid onto the surface of CMC-CSAC

Where *T* was the temperature (K), *R* was the gas constant (8.314 J/mol K), *ΔG* was the Gibbs free energy (kJ/mol), *ΔH* was the enthalpy (kJ/mol) and *ΔS* was the entropy (kJ/mol K). Figure 9 depicted the van’t Hoff plot and the enthalpy and entropy of adsorption were determined from the plot (Table 2). The negative values of both enthalpy and entropy confirmed that the adsorption process was exothermic. This could be further explained by the attraction of the adsorbate molecules onto the adsorbent surface. This resulted in the release of energy and, therefore, the heat of adsorption became negative (Foroutan et al. 2018). Because this process was exothermic, the entropy of the system decreased as the atoms were more ordered (Radoor et al. 2020). The positive Gibbs free energy indicated that the adsorption of both metal ions and lactic acid at all temperatures studied was not spontaneous.

**Table 2:** Thermodynamic properties of the adsorption of metal ions and lactic acid under different operating temperatures

Chemical species	*ΔH*, kJ/mol	*ΔS*, kJ/mol K	*ΔG* at *T* (K), kJ/mol		
			298	313	323
Metal ions	− 18.8179	− 0.0651	0.5842	1.5608	2.2119
Lactic acid	− 29.9296	− 0.1054	1.4711	3.0518	4.1055

### Adsorption isotherm

**Fig. 10:**
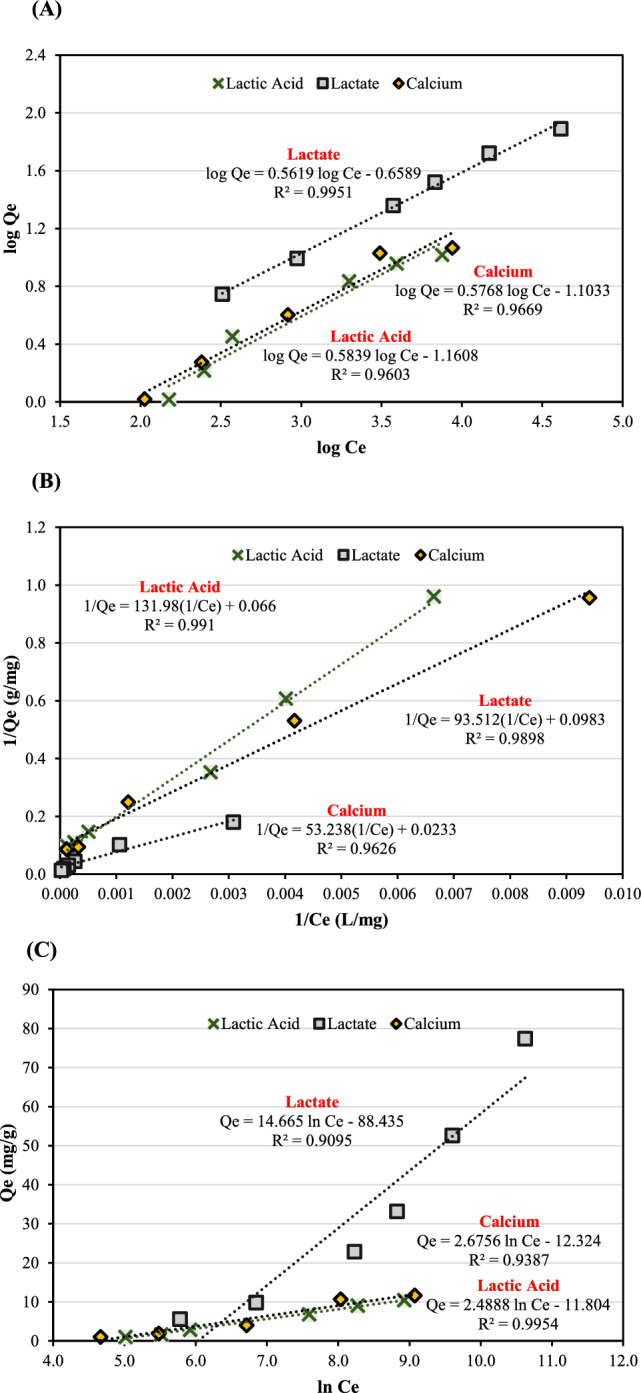
Linear fit of the **A** Freundlich, **B** Langmuir, and **C** Temkin isotherm models for free lactic acid, calcium, and lactate adsorbed onto CMC-CSAC. The CMC-CSAC adsorbent was prepared by grafting 5 g raw CSAC with 10 mL solution containing 2% CMC and 20% CA for 16 h. The batch adsorption was carried out at pH 6, room temperature with a 10:1 ratio of the solution to the adsorbent loading

The adsorption mechanism of metal ions and lactic acid onto the surface of CMC-CSAC was investigated by the adsorption equilibrium models, the characterization of CMC-CSAC before and after adsorption, and the density functional theory (DFT) calculation. The isotherm showed the relationship between the adsorbate concentration in the liquid solution at equilibrium and the amount that was adsorbed onto the solid adsorbent at a certain temperature. The isotherms were introduced to model the equilibrium adsorption data to investigate the adsorption mechanisms, the maximum adsorption capacity, and the properties of the adsorbents (Wang and Guo 2020). In this study, 3 isotherms were used for linearized data fitting based on their theoretical derivation and physical implication as an empirical model, chemical adsorption model, and physical adsorption model (Fig. 10). The adsorption empirical isotherm, such as the Freundlich model, lacked specific physical implication. On the other hand, the chemical isotherm-like Langmuir model described the monolayer adsorption process while the physical isotherm-like Temkin model explained the multilayer adsorption (Yang et al. 2019). The model parameters and the regression coefficients (*R*^*2*^) were described and compared in Table 3.

**Table 3:** Isotherm model parameters obtained from linear fitting of adsorption kinetics of free lactic acid, calcium, and lactate onto CMC-CSAC

Components	Free lactic acid	Calcium	Lactate
Freundlich isotherm			
* K*_*F*_ (L^1/n^mg^1−1/n^/g)	0.0691	0.0788	0.2193
* n*	1.7126	1.7337	1.7797
* 1/n*	0.5839	0.5768	0.5619
* R*^*2*^	0.9603	0.9669	0.9951
Langmuir isotherm			
* Q*_*m*_ (mg/g)	15.1515	1.0389	10.1729
* K*_*L*_ (L/mg)	0.0005	0.0181	0.0011
* R*^*2*^	0.9910	0.9626	0.9898
* R*_*L*_	0.1890–0.8870	0.0056–0.2078	0.0226–0.5193
Temkin isotherm			
* A* (L/mg)	0.0087	0.0100	0.0024
* b* (J/mol)	1079.0027	1003.6710	183.1178
* R*^*2*^	0.9954	0.9387	0.9095

The linear fit of the Freundlich isotherm model is shown in Fig. 10A. The Freundlich model is typically used to represent nonlinear adsorption. The constant *K*_*F*_ values of free lactic acid and calcium were 0.0691 L^1/n^mg^1−1/n^/g and 0.0788 L^1/n^mg^1−1/n^/g, respectively, while the *K*_*F*_ value of lactate was approximately 3 times larger than those of free lactic acid and calcium. The value of the adsorption intensity (*n*) of free lactic acid, calcium, and lactate were not much different. The values of *1/n* in between 0 and 1 represented the surface heterogeneity where the adsorption became more heterogeneous when *1/n* approached 0 (Table 3). The adsorption model becomes linearized when *n* = 1. The *R*^*2*^ values for all components adsorbed onto the surface of CMC-CSAC were greater than 0.96.

Figure 10B showed the linear fit of the Langmuir isotherm model. The maximum monolayer adsorption capacity (*Q*_*m*_) of free lactic acid was the highest (15.1515 mg/g CMC-CSAC), followed by that of lactate (10.1729 mg/g CMC-CSAC). The *Q*_*m*_ of calcium was significantly lower than those of free lactic acid and lactate. The separation factor (*R*_*L*_) was calculated by Eq. (10).

10 $${R}_{L}=\frac{1}{1+{K}_{L}{C}_{O}}$$

The values of* R*_*L*_ > 1, *R*_*L*_ = 1, and *R*_*L*_ < 1 revealed that the adsorption was unfavorable, linear, and favorable, respectively. From Table 3, the values of *R*_*L*_ for all components adsorbed onto the surface of CMD-CSAC were less than 1; therefore, the adsorption of free lactic acid, calcium, and lactate were favorable. The correlation coefficients for all components studied approaching 1 (*R*^2^ > 0.96) confirmed that the Langmuir model fit the experimental data well.

The Temkin isotherm model reveals the adsorption potential of the adsorbent to the adsorbates. Unlike the Langmuir isotherm model, this model postulates that adsorption is a multilayer process. Figure 10C shows the linear fit of the Temkin model. The model constants A and b are shown in Table 3. Both A and b values of free lactic acid and calcium adsorption were similar while those of lactate adsorption were much smaller. The positive values of the constant b revealed the endothermic adsorption (Al-Ghouti and Da’ana 2020). The *R*^*2*^ values of calcium and lactate adsorption were lower than 0.96, indicating that the Temkin model did not represent calcium and lactate adsorption onto the surface of CMC-CSAC well.

### Morphological characterization confirmed chemical adsorption model

**Fig. 11:**
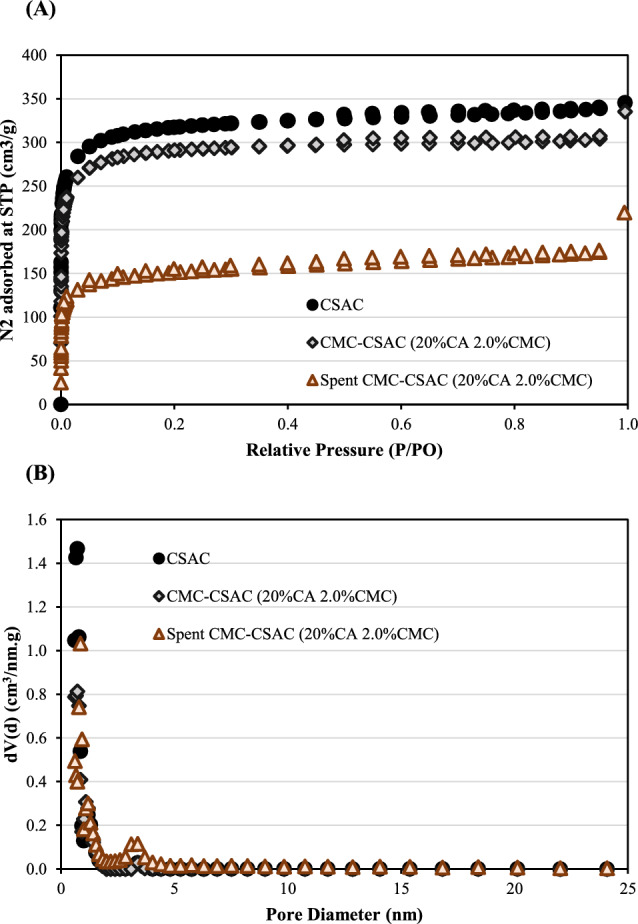
Morphological characteristics of CSAC adsorbents **A** nitrogen adsorption–desorption isotherms and **B** pore size distribution

Nitrogen adsorption was an experimental technique based on equilibrium Van der Waals interactions between N_2_ molecules and solid particles of CSAC adsorbents that quantified the specific surface area, pore size distribution, and pore volume of CSAC adsorbents. The Brunauer–Emmett–Teller theory (BET) was introduced to estimate the specific surface area that extended the Langmuir monolayer molecular adsorption model to a multilayer model using an equilibrium adsorption isotherm measured at the normal boiling point of N_2_ (77 K). Figure 11A depicted the Langmuir N_2_ adsorption–desorption isotherm of CSAC adsorbents, i.e., the raw CSAC, the grafted CMC-CSAC, and the spent CMC-CSAC. It was claimed that materials such as zeolites and some types of activated carbon with mainly narrow micropores followed the Langmuir isotherm model, where the pores filled at a very low relative pressure with a steep uptake due to the considerable adsorbent-adsorbate interactions (Bardestani et al. 2019). This was confirmed by the narrow steep curve shown in Fig. 11B and the DFT pore size reported in Table 4. The micropores of the CSAC adsorbents in this study were classified as medium-sized micropores (0.7 nm < DFT pore size < 0.9 nm). It should be noted that the specific surface area, pore size, and total pore volume remained unchanged after grafting the raw CSAC with CA and CMC (Berber 2020; Sepahvand et al. 2020). However, after adsorption, those values were decreased, indicating that ions were adsorbed onto the surface of the grafted CMC-CSAC (Table 4).

**Table 4:** Morphological properties of CSAC adsorbents

Sample	BET area (m^2^/g)	DFT pore size (nm)	Total pore volume (cm^3^/g)
CSAC	1191	0.72	0.48
CMC-CSAC (20% CA, 2.0% CMC)	1166	0.72	0.44
Spent CMC-CSAC (20% CA, 2.0% CMC)	573.3	0.85	0.05
Calcined spent CMC-CSAC (20% CA, 2.0% CMC)	1200	0.67	0.50

### Process comparison

Adsorption plays significant role in lactic acid recovery and purification. In the typical adsorption process, the adsorbents, including resins, zeolite, and activated carbon, were used for adsorbing lactic acid from the fermentation broth. Weak anionic ion exchange resins such as Amberlite IRA-96 and Amberlite IRA-67 were favorable for lactic acid adsorption from the fermentation broth at the slightly acidic pH (pH 4.8) due to its high desorption percentage and subsequently high recovery percentage when compared with the strong anionic ion exchange resins like Amberlite IRA-900 and Amberlite IRA-400 (Moldes et al. 2003). It was reported that the recovery of lactic acid adsorbed onto the weak anionic ion exchange resins such as Amberlite IRA-67, Amberlite IRA-92, and Amberlite IRA-96 was significantly high at 100% and 75% when the adsorbed resins were desorbed by HCl and H_2_SO_4_, respectively (Moldes et al. 2003; Tong et al. 2004). In the conventional lactic acid recovery process, impurities that remained in the fermentation broth were removed before lactic acid recovery by ion exchange resins to ease the separation process (Lee et al. 2017). The remaining glucose in the fermentation broth was typically removed by nanofiltration (Alvarado-Morales et al. 2021; Zaini et al. 2019). Lately, some weakly polar ion exchange resin such as HD-06 was used to adsorb glucose from the fermentation broth before the adsorption of lactic acid and anions by the anionic ion exchange resins such as Lewaitit S3428 (Song et al. 2017). Meanwhile, the cationic ion exchange resins such as Lewatit S2568H was used to eliminate the cations or the metal ions from the fermentation broth (Gonzalez et al. 2006).

As mentioned previously, lactic acid recovery and purification from the fermentation broth typically consisted of multi-process steps that integrated the membrane separation with the adsorption (Lee et al. 2017). The recovery process usually started with the removal of cell biomass and suspended solids and colloids by microfiltration and ultrafiltration. Later, lactic acid recovery was conducted using ion exchange resins. Later, further purification was carried out by nanofiltration, where the organic molecules such as glucose were eliminated. The inorganic impurities were removed either by nanofiltration or ion exchange resins. This resulted in the final purity of this recovery and separation operation reached 99.5%. However, adsorption using the ion exchange resins had major drawbacks; for example, a large quantity of chemicals, acid, base, and water was required during the adsorption, washing, desorption, and regeneration processes (Gonzalez et al. 2006). The utilization of a large amount of chemicals, acid, base, and water resulted in a large amount of process effluent and wastewater treatment loading. In addition, the resin capacity (*Q*_*m*_) and the resin cost were the 2 major factors that contributed to the process cost (Alves De Oliveira et al. 2020; Din et al. 2021; Xu et al. 2018).

It was reported that zeolite and activated carbon adsorbed lactic acid; however, the desorption efficiency (also known as the recovery percentage) was only 63% when the spent adsorbents were desorbed by deionized water, but the recovery percentage was increased when the organic solvent (acetone) was used as the eluent (80% recovery) (Aljundi et al. 2005; Gao et al. 2011). The proper surface modification in this study revealed that the activated carbon could efficiently adsorb metal ions with a reasonable lactic acid recovery and purity in a single-stage batch adsorption. The adsorption process using CMC-CSAC prepared in this study was simple unlike the adsorption with the ion exchange resins that required resin pretreatment using acidic or alkali solution. The CMC-CSAC adsorbent adsorbed all metal ions present in the fermentation broth (Table 5). Metal ions in the fermentation broth comprised of both monovalent (K^+^ and Na^+^) and divalent (Ca^2+^, Mg^2+^, Mn^2+^, and Fe^2+^) ions. The hydrated radii (R_H_) of the monovalent ions were 0.33 nm for K^+^ and 0.36 nm for Na^+^ while the hydrated radii of the divalent ions were 0.41 nm for Ca^2+^, 0.43 nm for Mg^2+^, 0.44 nm for Mn^2+^, and 0.43 nm for Fe^2+^ (Nightingale 1959). Porous materials typically exhibit pore size-dependent adsorption. The microporous materials including the CMC-CSAC adsorbent (pore diameter of 0.72 nm) prepared in this study were, therefore, more suitable for adsorbing the monovalent and divalent ions than the multivalent ions (Gabelich et al. 2002; Seo et al. 2010). The highest adsorption capacity of Ca^2+^ onto the surface of CMC-CSAC among other metal ions could be explained by its concentration effect together with the compatible pore diameter of CMC-CSAC and its hydrated radius (Xu et al. 2008).

**Table 5:** Ion uptake by the 16-h CMC-CSAC adsorbent

Components	Lactic acid	Ca^2+^	K^+^	Mg^2+^	Na^+^	Fe^2+^	Mn^2+^	Total metal ions
Fermentation broth (g/L)	100.5445	16.2484	0.1694	0.1855	0.0690	0.0192	0.0078	16.6993
After adsorption (g/L)	96.2908	14.3562	0.1468	0.1807	0.0658	0.0188	0.0074	14.7757
Percentage uptake (%)	4.23	11.65	13.34	2.59	4.64	2.08	5.12	11.52

The 16-h CMC-CSAC adsorbent (5 g) was soaked in 50 mL cell-free lactic acid fermentation broth (pH 6) at room temperature for 150 min

The typical adsorbent regeneration process utilizes a large amount of acid, alkali, enzyme, and water that eventually increases the wastewater treatment load. In this study, the low-cost spent CMC-CSAC adsorbent was subjected to calcination. The calcined spent CMC-CSAC adsorbent exhibited the excellent surface morphological properties and was therefore effectively used in CO_2_ adsorption (Table 4 and Additional file 1: Fig. S1). A substantial amount of metal ions especially calcium remained in the spent CMC-CSAC adsorbent after calcination acted as activating agents, leading to an increase in the total pore volume and the adsorption capacity of CO_2_ (Table 4 and Additional file 1: Fig. S1). The CMC-CSAC adsorbent was an organic material derived from the agricultural residues. It was effectively used for eliminating metal ions in lactic acid remained in the fermentation broth. Instead of the typical regeneration of the spent CMC-CSAC adsorbent, calcination was introduced and the calcined spent CMC-CSAC was further used in CO_2_ adsorption. Therefore, we claim that the utilization of CMC-CSAC in this study is considered economically and environmentally friendly.

## Conclusions

A single-stage adsorption by grafted nanostructured CMC-CSAC was used for partial recovery of lactic acid from the fermentation broth. Crosslinking CSAC with CMC by CA enhanced the surface chemistry while the morphology remained unchanged; therefore, this improved metal ion adsorption with a relatively low loss of lactic acid. The pH of the solution significantly affected lactic acid recovery. The optimal operating conditions were at room temperature and pH 6 with a 10:1 ratio of liquid solution to the adsorbent loading. A thermodynamic study revealed that the adsorption process was exothermic and non-spontaneous. The Langmuir isotherm model explained the adsorption phenomena. The findings in this study support bioresource processing and utilization in a sustainable manner. The recovery of lactic acid from the metal ions remained in the fermentation broth using the CMC-CSAC bioadsorbent was simple. Unlike the polymeric ion exchange resins, the adsorbent preparation and recovery steps can be omitted and the spent CMC-CSAC can be simply landfilled. Therefore, this process is environmentally friendly.

### Supplementary Information

**Additional file 1: Table S1.** The components remained in lactic acid fermentation broth obtained from different batch cultivation used for metal ion removal by CMC-CSAC. **Fig. S1.** CO_2_ adsorption of calcined spent CMC-CSAC at room temperature.

## Data Availability

All data generated or analyzed during this study are included in this article.

## Abbreviations

CSAC: Coconut shell activated carbon
CMC: Carboxymethyl cellulose
CA: Citric acid
CMC-CSAC: Carboxymethyl cellulose
CaLA: Calcium lactate
LA: Lactic acid
DI: Deionized
R_H_: Hydrated radius
