# Supplementary material for: Metal ion removal using a low-cost coconut shell activated carbon bioadsorbent in the recovery of lactic acid from the fermentation broth
Source: Bioresour Bioprocess. 2023 Sep 1;10(1):58. doi: 10.1186/s40643-023-00672-1 (PMC10992777; doi:10.1186/s40643-023-00672-1)
Supplement: Supplementary file 1 — Additional file 1: Table S1. The components remained in lactic acid fermentation broth obtained from different batch cultivation used for metal ion removal by CMC-CSAC. Fig. S1. CO2 adsorption of calcined spent CMC-CSAC at room temperature. [file 40643_2023_672_MOESM1_ESM.docx]

**Table S1.** The components remained in lactic acid fermentation broth obtained from different batch cultivation used for metal ion removal by CMC-CSAC.

| Study factors | Analyzed by HPLC | | Analyzed by AA | | | | | |
| --- | --- | --- | --- | --- | --- | --- | --- | --- |
|  | Lactic acid | Glucose | Ca^2+^ | K^+^ | Mg^2+^ | Na^+^ | Fe^2+^ | Mn^2+^ |
| CMC and CA concentrations | 76.07 | 0.00 | 13.65 | 0.60 | 0.24 | 0.05 | 0.01 | 0.01 |
| Grafting time | 83.31 | 0.00 | 15.94 | 0.56 | 0.24 | 0.04 | 0.01 | 0.01 |
| pH | 100.54 | 0.00 | 16.25 | 0.17 | 0.19 | 0.07 | 0.02 | 0.01 |
| Contact time | 90.00 | 5.63 | 19.53 | 0.23 | 0.19 | 0.04 | 0.02 | 0.01 |
| Ratio of liquid broth to CSAC | 80.40 | 26.82 | 16.25 | 0.17 | 0.19 | 0.07 | 0.02 | 0.01 |
| Thermodynamic study | 91.57 | 5.68 | 20.25 | 0.23 | 0.18 | 0.04 | 0.02 | 0.01 |

**Fig. S1.** CO_2_ adsorption of calcined spent CMC-CSAC at room temperature.
